# Deadwood-Inhabiting Bacteria Show Adaptations to Changing Carbon and Nitrogen Availability During Decomposition

**DOI:** 10.3389/fmicb.2021.685303

**Published:** 2021-06-17

**Authors:** Vojtěch Tláskal, Petr Baldrian

**Affiliations:** Laboratory of Environmental Microbiology, Institute of Microbiology of the Czech Academy of Sciences, Praha, Czechia

**Keywords:** deadwood, bacterial genomes, cellulose, mycophagy, nitrogen fixation

## Abstract

Deadwood decomposition is responsible for a significant amount of carbon (C) turnover in natural forests. While fresh deadwood contains mainly plant compounds and is extremely low in nitrogen (N), fungal biomass and N content increase during decomposition. Here, we examined 18 genome-sequenced bacterial strains representing the dominant deadwood taxa to assess their adaptations to C and N utilization in deadwood. Diverse gene sets for the efficient decomposition of plant and fungal cell wall biopolymers were found in *Acidobacteria, Bacteroidetes*, and *Actinobacteria*. In contrast to these groups, *Alphaproteobacteria* and *Gammaproteobacteria* contained fewer carbohydrate-active enzymes and depended either on low-molecular-mass C sources or on mycophagy. This group, however, showed rich gene complements for N_2_ fixation and nitrate/nitrite reduction—key assimilatory and dissimilatory steps in the deadwood N cycle. We show that N_2_ fixers can obtain C independently from either plant biopolymers or fungal biomass. The succession of bacteria on decomposing deadwood reflects their ability to cope with the changing quality of C-containing compounds and increasing N content.

## Introduction

The decomposition of deadwood in forests is a complex process during which plant polymers such as cellulose, hemicellulose, and lignin are exploited by members of the microbial community. Since the deadwood microbiome is dominated by fungi, their cell wall constituents represent another important resource, especially in the late phases of decomposition. Deadwood is estimated to contain 8% of the total global forest carbon (C) stock (Pan et al., 2011; Martin et al., 2021), making it an important source of carbon. However, the degree of the recalcitrance of C in deadwood components varies considerably and changes over time with the progress of decomposition. Fungi and bacteria produce a wide array of enzymes to access complex C sources, typically termed carbohydrate-active enzymes (CAZymes, Berlemont and Martiny, 2015). C-source niche partitioning in the utilization of C compounds is observed among microbial taxa (Gallardo et al., 2021), as well as various levels of opportunism in C acquisition (Lladó et al., 2019), where released degradation products may be utilized by microbes incapable of decomposition without the need to invest in enzyme production (Berlemont and Martiny, 2013). In contrast to C, the initial nitrogen (N) content in deadwood is extremely low (typically <1%), which leads to strong N limitation of microbial growth and substrate colonization (Tláskal et al., 2017). N accumulates in deadwood during decomposition either due to transport via the mycelium of ectomycorrhizal and soil foraging fungi (Philpott et al., 2014) or via the activity of the diazotrophic bacterial community (Rinne et al., 2016; Tláskal et al., 2021a). Such an increase gradually alleviates N limitation, leading to a more favorable C:N ratio of the whole habitat (Weedon et al., 2009).

A previous study analyzing the decomposition of *Fagus sylvatica* deadwood through metatranscriptome analysis showed the major role of fungi in C release, as demonstrated by their dominance in CAZyme production (Tláskal et al., 2021a). Notably, even the composition of the bacterial community in deadwood is heavily affected by fungi due to their primary access to C resources (Odriozola et al., 2021). The effect of fungi on bacteria might be direct (such as through antibiosis) or indirect through substrate acidification, allowing for the selection of acid-tolerant bacterial taxa (Valášková et al., 2009; Christofides et al., 2019; Johnston et al., 2019). Fungal biomass formation also represents a nutritional basis for several bacteria (Brabcová et al., 2016; López-Mondéjar et al., 2018). Such mycophagous bacteria developed enzymes for chitin degradation to overcome N limitation by feeding on fungal biomass containing N-rich chitin and chitosan (Uroz et al., 2013; Brabcová et al., 2016; Starke et al., 2020). As a result of fungal modulation and changing conditions in the substrate, the composition and abundance of the bacterial community in deadwood change over time showing an increase of bacterial biomass and taxa succession toward community similar to that in soil (Rinta-Kanto et al., 2016; Tláskal et al., 2017). Among the factors influencing bacterial community composition in deadwood, the most often reported are pH, the C:N ratio, and water content (Folman et al., 2008; Hoppe et al., 2015; Tláskal et al., 2017; Moll et al., 2018; Mieszkin et al., 2021), but the biopolymer composition and fungal abundance are also important (Rinta-Kanto et al., 2016; Odriozola et al., 2021). Due to limited knowledge of the functional traits of deadwood bacteria, it is unclear how exactly these factors influence the members of the bacterial community or vice versa and which adaptations make bacterial species successful at various stages of deadwood decomposition.

While culture-independent methods may provide the best picture of bacterial community composition and gene expression, the characterization of isolates of dominant bacteria represents the best approach for the characterization of their phenotypes and traits (Lladó et al., 2016). The genome sequencing of bacterial isolates has helped distinguish the functional guilds of decomposers and opportunists within soil bacteria (Lladó et al., 2019). While metagenome analysis may help predict bacterial traits based on the construction of metagenome-assembled genomes (MAGs, Tláskal et al., 2021a), this approach has multiple limitations. MAGs are mostly incomplete, subject to potential contamination by non-target sequences, and often difficult to link to existing 16S rRNA gene sequences and, as such, exact bacterial identities (Bowers et al., 2017). Furthermore, the ability to recover dominant bacteria as MAGs is also variable (Nayfach et al., 2020). All of these limitations can be overcome by isolate characterization, which also offers the opportunity for phenotype screening in cultures (Madin et al., 2020).

The present study isolated the dominant bacteria from decomposing deadwood to describe their functional traits through genome sequencing and phenotype analysis and used this information to explain the preference of specific bacterial taxa for certain stages of deadwood decomposition. We focused on the bacterial ability to release C from plant and fungal polymers and bacterial assimilatory and dissimilatory N cycling pathways. We expect dominant bacteria to show differing occurrences throughout deadwood decomposition stages, driven by substrate availability and their metabolic ability to be constrained by taxonomic identity.

## Materials and Methods

### Strain Isolation

This study was conducted in the Žofínský Prales National Nature Reserve, a temperate unmanaged forest in the south of the Czech Republic (48°39′57″N, 14°42′24″E). The core zone of the forest reserve (42 ha) has never been managed, and any human intervention ceased in 1838 when it was declared a reserve. This reserve thus represents a rare fragment of European temperate virgin forest with deadwood left to spontaneously decompose. The reserve is situated at 730–830 m above sea level and the bedrock is almost homogeneous and consists of finely to medium-grainy porphyritic and biotite granite. The annual average rainfall is 866 mm, and the annual average temperature is 6.2°C (Anderson-Teixeira et al., 2015). At present, the reserve is covered by a mixed forest where *Fagus sylvatica* predominates in all diameter classes (51.5% of the total living wood volume), followed by *Picea abies* (42.8%) and *Abies alba* (4.8%). The mean living tree volume is 690 m^3^ h^−1^, and the volume of coarse woody debris (logs, represented by tree trunks and their fragments) ranges from 102 to 310 m^3^ h^−1^ with an average of 208 m^3^ h^−1^ (Král et al., 2010; Šamonil et al., 2013). Logs are repeatedly surveyed, and the approximate age of each log, the cause of death (stem breakage, windthrow, etc.), and status before downing (fresh, decomposed) is known.

Decomposing wood samples were collected as described previously (Tláskal et al., 2017). Briefly, dead tree trunks (coarse woody debris; CWD) of *F. sylvatica, P. abies*, and *A. alba* with breast height diameters between 30 and 100 cm at the time of downing were chosen to evenly represent all decay lengths (age classes) of deadwood and the span of the diameter range. Trees decomposing as standing snags were omitted to exclude CWD with unclear decay lengths. The CWD decay length ranged from <5 to >38 years. In October 2013, four samples were obtained by drilling with an electrical drill vertically covering sapwood and heartwood along the whole decomposing stem. Part of the material was used for the description of fungal and bacterial communities and wood chemistry (Baldrian et al., 2016; Tláskal et al., 2017), while another part was used for the isolation of bacteria. For this latter category of subsamples, wood chips from each stem were pooled together and transported to the laboratory, where they were cooled until the next day. Approximately 2 g of wood material were shaken with 15 mL of Ringer solution for 2 h and this suspension was diluted 10^4^–10^6^×. Five hundred microlitres of each dilution were plated on nutrient-limited NB medium (0.26 g L^−1^ nutrient broth, 15 g L^−1^ agar, pH 5) and WYA4 medium (0.1 g L^−1^ yeast extract, 15 g L^−1^ agar, pH 5, Valášková et al., 2009) with cycloheximide (50 mg L^−1^). Occurrences of bacterial colonies were recorded on Petri dishes incubated at 24°C for 8 weeks and marked with a marker indicating the week of the first appearance. Colony PCR was used to infer the taxonomy of the selected strains that appeared during the course of incubation. PCR premix and cycling conditions were as follows: 2.5 μL of 10× buffer for DyNAzyme DNA Polymerase; 0.75 μL of DyNAzyme II DNA polymerase (2 u μL^−1^); 0.75 μL of BSA (20 mg mL^−1^); 0.5 μL of PCR Nucleotide Mix (10 mM); 1 μL of primer eub530f (10 μM, 5′-GTG CCA GCM GCN GCG G); 1 μL of primer eub1100br (10 μM, 5'-GGG TTN CGN TCG TTG, Lane, 1991) and sterile ddH_2_O up to 25 μL, with amplification started at 94°C for 5 min followed by 35 cycles of 94°C for 1 min, 62°C for 1 min, 72°C for 1 min and a final setting of 72°C for 10 min. Sanger sequencing was performed from the reverse primer as an external service. The obtained sequences were compared by BLASTn with bacterial 16S rRNA-based community data from the same deadwood objects that were used for isolation (Tláskal et al., 2017). Bacterial strains with high similarity (>97%) and coverage (>90%) relative to the most abundant deadwood OTUs were selected for further cultivation. The strains that were cultivable on laboratory media in subsequent passages and those that exhibited sufficient growth and biomass production were used for genome sequencing and culturing tests. By this approach, we were able to select 18 bacterial strains with high similarity to highly abundant bacterial OTUs from the studied deadwood.

### Cultivation Tests

The activity of the cell wall-associated fraction of enzymes was measured in cells after 2 weeks of incubation in triplicate in 50 mL of liquid GY-VL55 medium as described previously (Lasa et al., 2019). To assess the ability to grow on cellulose, carboxymethyl cellulose medium (CMC) was prepared (5 g L^−1^ CMC, 2 g L^−1^ yeast extract, 15 g L^−1^ agar, pH 5). Plates were incubated at 24°C for 2 weeks. After incubation, 12 mL of 1% Congo Red was poured onto each plate, staining was performed for 30 min, the Congo Red was replaced with 12 mL of 1 M NaCl, the NaCl was left for 30 min, the NaCl was replaced with 12 mL of H_2_O, and the H_2_O was left overnight. The presence of a halo around the colonies indicated CMC utilization.

### DNA Extraction, Sequencing, Genome Assembly, and Annotation

Selected bacterial strains were cultivated in 50 mL of GY-VL55 liquid medium shaken in Erlenmeyer flasks (Lladó et al., 2019) for 2 weeks at 23°C. Cells were collected by centrifugation, and DNA was extracted by an ArchivePure DNA Yeast and Gram-+ Kit (5 Prime, Germany) according to the manufacturer's instructions. DNA was sheared by a Bioruptor Pico sonication device (Diagenode, Belgium) to an average length of 550 bp, and sequencing adapters were ligated by the TruSeq DNA PCR-Free Library Prep Kit (Illumina Inc., USA). Ligated libraries were sequenced in-house on the Illumina MiSeq platform.

Genome assembly of each isolate was performed using a Unicycler 0.4.7 (Wick et al., 2017) in normal mode with the following programs: SPAdes 3.13.0 (Bankevich et al., 2012), BLAST 2.7.1+ (Altschul et al., 1997), Bowtie 2.2.4 (Langmead et al., 2009), SAMtools 1.9 (Li et al., 2009), and Pilon 1.22 (Walker et al., 2014). Prokka 1.13.3 (Seemann, 2014) with RNAmmer (Lagesen et al., 2007) was used for gene calling, annotation, and rRNA gene identification. The taxonomy of the closest complete genome in the NCBI database with >97% similarity of 16S rRNA gene sequences were used to assign strains at the genus level. GToTree v1.5.39 (Lee, 2019) together with Prodigal (Hyatt et al., 2010), HMMER3 (Eddy, 2011), Muscle (Edgar, 2004), trimAI (Capella-Gutiérrez et al., 2009), FastTree2 (Price et al., 2010), and GNU Parallel (Tange, 2018) was used to construct a phylogenetic tree of the genomes based on a set of HMM profiles for 74 bacterial single-copy genes.

For CAZyme annotation, Prokka gene prediction was used to obtain the sequences of genes. Amino acid sequences were compared with the dbCAN database version 07312018 using the run-dbcan.py script (Zhang et al., 2018) and HMMER 3.2.1 (Eddy, 2011). CAZymes with an e-value ≤ 1E^−30^ were considered for further annotation and their most probable target substrates were identified based on the genes with characterized functions in the CAZy database (http://www.cazy.org; Lombard et al., 2014). Functions of predicted genes were annotated with the hmmsearch function in HMMER 3.2.1, using the FOAM database as a source of HMMs for relevant genes (Prestat et al., 2014). FOAM functions with an e-value ≤ 1E^−30^ were considered for further annotation. In the case of genes with more than one hit in the dbCAN and the FOAM databases, the function with the lowest e-value was selected.

### Metagenome Mapping

The metagenomes of naturally decomposing *F. sylvatica* deadwood were obtained in a previous study (Tláskal et al., 2021a, Tláskal et al., under review) and used to quantify genome coverage in deadwood of various decay lengths. This metagenome study included 25 CWD samples of *F. sylvatica*. The CWD samples were classified into five decay length classes of <4, 4–7, 8–19, 20–41, and > 41 years. Metagenomic reads were mapped to genomes as described previously (Lladó et al., 2019) using Bowtie v2.3.4.1 (Langmead and Salzberg, 2013). Metagenomic reads from sample with NCBI accession SRR10968255 were omitted from mapping due to under-sampling. The number of mapped reads was normalized to the sequencing depth of each sample, divided by the genome length of individual strains, and multiplied by the average genome size of sequenced bacteria. Differences among groups were tested by the Kruskal-Wallis test using the agricolae package (de Mendiburu, 2017) and considered to be statistically significant at the level *P* < 0.05.

### 16S rRNA Gene-Based Phylogeny of OTUs and Strains

Dereplicated 16S rRNA genes of strains with sequenced genomes were merged with representative OTU sequences (most frequent sequences from each OTU, Tláskal et al., 2017). Only the globally most abundant OTUs represented by an abundance >0.5% in >10 samples were selected (*n* = 86 OTUs). Sequences were aligned by the MAFFT online tool (Katoh et al., 2018) with default settings. A phylogenetic tree was constructed using FastTree2 (Price et al., 2010) with default settings. The taxonomy of the tree tips was assigned with DECIPHER v2.0 (Wright, 2016) using SILVA SSU database release 138 (Quast et al., 2013). A phylogenetic tree of 16S rRNA sequences from all 959 obtained isolates was constructed by the same approach. The diversity of the whole isolated collection was inferred by clustering using VSEARCH 2.4.3 (Rognes et al., 2016). Tables were processed using R 4.0.2 (R Core Team, 2020) and tidyverse 1.3.0 (Wickham et al., 2019). The resulting images were processed with Inkscape 1.0 (https://inkscape.org/).

## Results

Isolation yielded 959 isolates, with *Gammaproteobacteria* being the most represented class, followed by the class *Alphaproteobacteria* and the phyla *Actinobacteria, Bacteroidetes, Acidobacteria*, and *Firmicutes* (Supplementary Figure 1A). The largest share of isolates was represented by the *Burkholderia-Caballeronia-Paraburkholderia* complex (17%), followed by the genera *Mucilaginibacter* (7%) and *Pseudomonas* (7%, Supplementary Figure 1B). Isolates clustered into 467 OTUs (>99% similarity) and were binned to 4.5% of OTUs from the 16S rRNA amplicon study from the same CWD samples published previously (Tláskal et al., 2017). The isolates represented on average 33.5 ± 1.3% of bacteria in a CWD sample. The youngest age class of deadwood, comprising CWD samples with a decay length <5 years, was most represented by the isolates (38.8 ± 2.6%).

**Figure 1:**
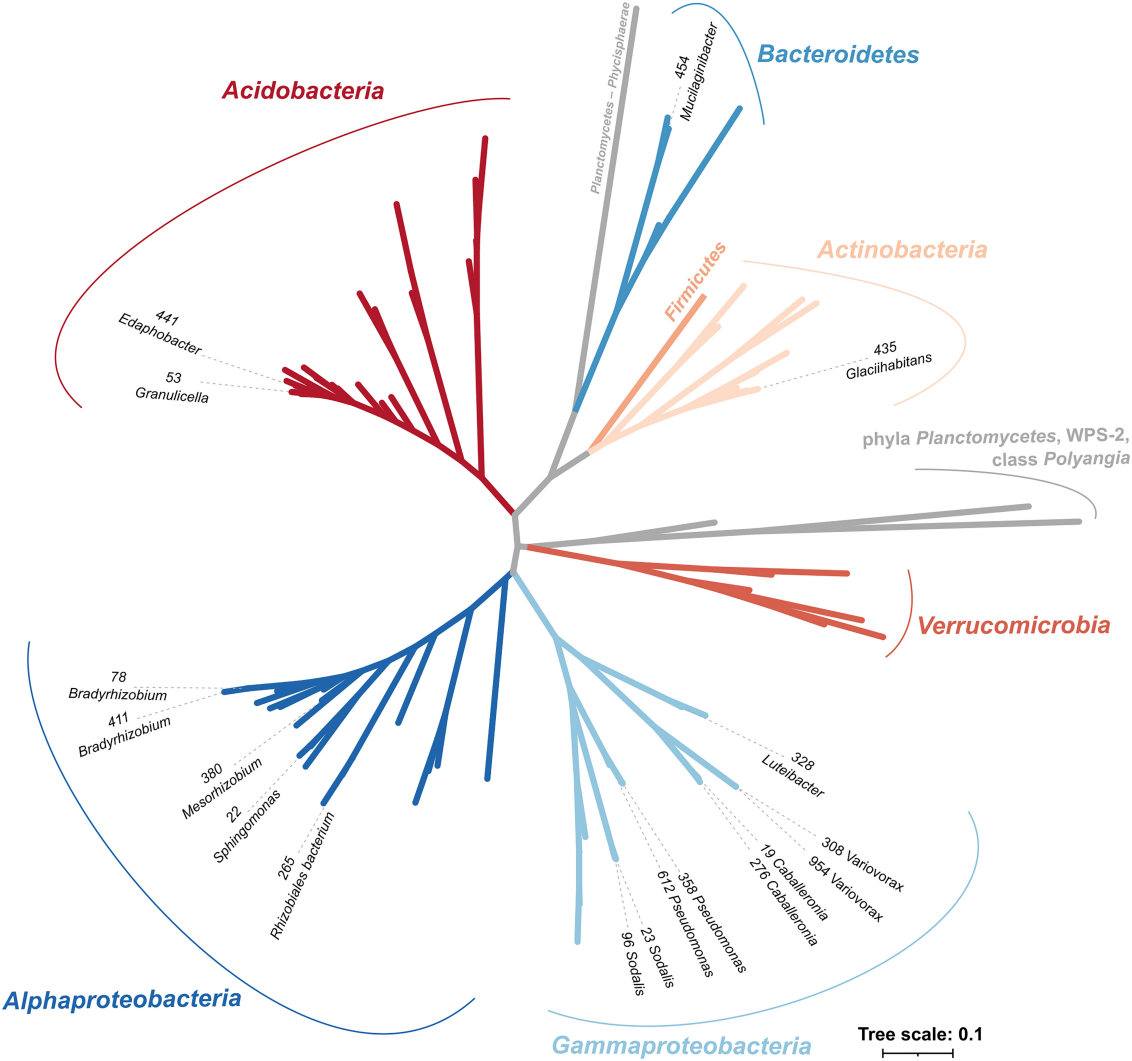
Phylogenetic tree of strains with sequenced genomes in the context of representative sequences of the 87 most abundant OTUs from *Fagus sylvatica, Picea abies*, and *Abies alba* deadwood based on the V4 region of 16S rRNA gene. Strains are denoted with strain IDs and genus names and cover the majority of the dominant bacterial phyla in deadwood. Phyla are labeled with different colors, and taxonomy follows the SILVA 138 database. The most abundant bacteria are based on the study of Tláskal et al. (2017).

The selected sequenced strains binned to 0.7% of bacterial OTUs, representing 15.3 ± 0.8% of bacteria in CWD samples (Supplementary Figure 2; Table 1). The phylogeny of 86 dominant OTUs in the whole amplicon dataset, i.e., those representing >0.5% sequences in >10 CWD samples, showed that we retrieved members of the top five deadwood bacterial phyla and classes of *Proteobacteria*. These were *Acidobacteria, Alphaproteobacteria, Gammaproteobacteria, Actinobacteria*, and *Bacteroidetes* (Figure 1; Table 1). Members of other phyla that represented only a small minority among the dominant OTUs–*Firmicutes* (*n* = 1), class *Polyangia* (*n* = 1), *Planctomycetes* (*n* = 2), *Verrucomicrobia* (*n* = 5), and WPS-2 (*n* = 1)–either were not isolated or failed to grow in subsequent cultures. Except for the strains of *Bradyrhizobium* (1.5%), each genome represented on average <1% of bacteria across all CWD samples. Up to 26.7% of bacteria were represented in the best-covered CWD samples (Table 1).

**Table 1:** Genomic traits of dominant bacteria associated with decomposing deadwood.

**Strain ID**	**Taxonomy**	**Source medium**	**CMC growth**	**Mean abundance in 16S rRNA data (%)**	**Max abundance in 16S rRNA data (%)**	**Assembly size (Mbp)**	**GC content (%)**	**Gene number**	**Accession**	**SRA**
435	*Glaciihabitans*; *Actinobacteria*	WY		0.60 ± 0.09	10.80	3.7	65.9	3600	PRJNA613978	SRR11413040
454	*Mucilaginibacter*; *Bacteroidetes*	WY		0.16 ± 0.05	18.38	4.7	43.2	4112	PRJNA613980	SRR11413148
53	*Granulicella*; *Acidobacteria*	NB		0.65 ± 0.05	11.69	6.1	57.7	5062	PRJNA613883	SRR11396417
441	*Edaphobacter*; *Acidobacteria*	WY		0.73 ± 0.08	10.32	4.9	58.8	4133	PRJNA613979	SRR11413017
22	*Sphingomonas*; *Alphaproteobacteria*	NB		0.35 ± 0.07	16.74	4.4	66.8	3947	PRJNA613993	SRR11396416
265	*Rhizobiales* b.; *Alphaproteobacteria*	WY		0.31 ± 0.02	2.95	4.2	64.1	3934	PRJNA613888	SRR11412665
380	*Mesorhizobium*; *Alphaproteobacteria*	WY		0.09 ± 0.02	0.42	6.9	62.6	6578	PRJNA701764	SRR13706190
78	*Bradyrhizobium*; *Alphaproteobacteria*	NB	yes	1.51 ± 0.12	10.01	6.0	62.1	5585	PRJNA613884	SRR11412340
411	*Bradyrhizobium*; *Alphaproteobacteria*	WY	yes	1.51 ± 0.12	10.01	7.3	61.1	6712	PRJNA613977	SRR11413018
276	*Caballeronia*; *Gammaproteobacteria*	WY		0.46 ± 0.06	6.68	6.8	59.7	6165	PRJNA613889	SRR11413014
19	*Caballeronia*; *Gammaproteobacteria*	NB	yes	0.46 ± 0.06	6.68	9.1	60.0	8229	PRJNA613879	SRR11392425
954	*Variovorax*; *Gammaproteobacteria*	NB		0.16 ± 0.02	2.76	8.1	66.1	7409	PRJNA613983	SRR11413163
308	*Variovorax*; *Gammaproteobacteria*	WY		0.16 ± 0.02	2.76	7.9	66.0	7144	PRJNA615766	SRR11432075
328	*Luteibacter*; *Gammaproteobacteria*	WY		0.77 ± 0.28	19.95	5.1	64.2	4532	PRJNA613890	SRR11413015
612	*Pseudomonas*; *Gammaproteobacteria*	WY		0.32 ± 0.12	26.70	7.4	59.7	6455	PRJNA613981	SRR11413149
358	*Pseudomonas*; *Gammaproteobacteria*	WY	yes	0.48 ± 0.18	13.88	5.5	62.0	5049	PRJNA613975	SRR11413016
96	*Sodalis*; *Gammaproteobacteria*	NB	yes	0.30 ± 0.08	10.61	5.9	54.0	5307	PRJNA613885	SRR11431239
23	*Sodalis*; *Gammaproteobacteria*	NB	yes	0.30 ± 0.08	10.61	6.4	55.0	5770	PRJNA599932	SRR10875134

*The average and maximal representations in the CWD are based on the 16S rRNA gene community data (Tláskal et al., 2017)*.

**Figure 2:**
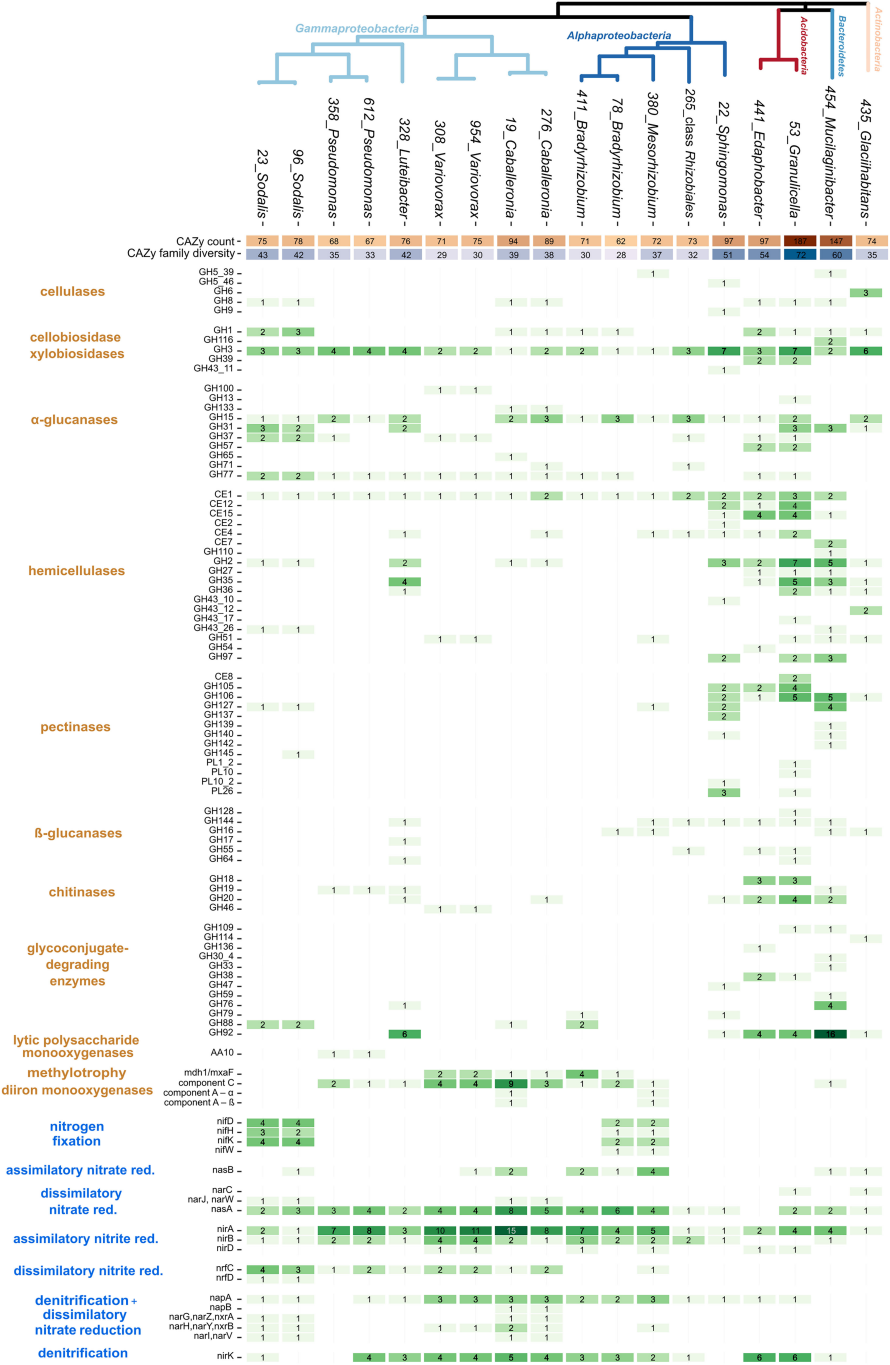
Genomic traits of dominant bacteria associated with decomposing deadwood. Heatmap shows CAZy counts, counts of genes for low-molecular-mass compounds utilization, and counts of N cycle-related genes together with putative functional annotation of each gene. The total number of CAZy genes and counts of CAZy families (CAZy diversity) per genome are depicted as heatmaps in the upper rows. A phylogenetic tree based on single-copy marker genes shows the phylogenetic relatedness of individual strains.

CAZy analysis showed a considerable level of variation in the carbon utilization traits among strains (Figure 2). Four strains were distinct in terms of high CAZyme counts and diversity: two *Acidobacteria* (*Edaphobacter* strain 441 and *Granulicella* strain 53), *Mucilaginibacter* strain 454, and *Sphingomonas* strain 22. The *Granulicella* strain showed the most CAZymes (*n* = 187 in 72 CAZy families), representing 3.7% of its total genes. In contrast, six members of the genera *Variovorax, Pseudomonas*, and *Bradyrhizobium* contained only 62–75 CAZymes in 28–35 CAZy families, representing 1% of their total genes (Figure 2).

Functional analysis of the identified CAZymes showed the specific C sources targeted by the strains (Figure 2). Genes for cellulose degradation were present in three copies of cellulase GH6 in the genome of *Glaciihabitans* strain 435. *Mucilaginibacter* strain 454 and *Sphingomonas* strain 22 each contained two copies of cellulases. Other strains with a single copy of a cellulase gene included both *Acidobacteria* strains, both *Caballeronia* strains, both *Sodalis* strains, and *Mesorhizobium* strain 380. An increased number of chitinase genes were present in *Granulicella* strain 53 and *Edaphobacter* strain 441 (*n* = 7 and 5, respectively). *Mucilaginibacter* strain 454 contained three genes for enzymes involved in chitin degradation, and *Luteibacter* strain 328 contained two chitinases. Both *Pseudomonas* strains encoded a single chitinase gene, and genes encoding enzymes targeting chitin were present in both *Variovorax* strains, *Caballeronia* strain 276 and *Sphingomonas* strain 22. An increased number of hemicellulases were present in two *Acidobacteria* strains, *Sphingomonas* strain 22, *Mucilaginibacter* strain 454, and *Luteibacter* strain 328. CAZymes for the labile substrates cellobiose and xylobiose and α-glucanases were present throughout the isolated strains without evident differences. CAZymes for other labile substrates, pectinases, β-glucanases, and glycoconjugate-degrading enzymes, were preferentially found in high-CAZy-containing *Acidobacteria* strains, *Mucilaginibacter* strain 454, *Glaciihabitans* strain 435, and *Sphingomonas* strain 22. Two *Pseudomonas* strains contained lytic polysaccharide monooxygenase genes from CAZy family AA10 for the oxidation of a broad range of polysaccharides. Some of the low-CAZy genomes contained genes for C uptake from alternative sources: the *mxaF* gene for methanol dehydrogenase, which oxidizes methanol, was present in *Variovorax* and *Caballeronia* (both *Burkholderiales*), and four copies of this gene were present in *Bradyrhizobium* strain 411. *Mesorhizobium* together with *Caballeronia* contained genes for soluble diiron monooxygenases.

*Luteibacter* strain 328 showed by far the most active cell wall-associated enzymatic fraction. Except for *Luteibacter*, chitinase activity was highest in *Bradyrhizobium* strains, *Variovorax* strains, and *Pseudomonas* strains. Strains of *Pseudomonas, Variovorax*, and *Luteibacter* were not able to grow on CMC medium but displayed cellobiohydrolase and β-glucosidase activities suitable for the degradation of cellulose-derived oligosaccharides (Table 1; Supplementary Figure 3).

Nitrogen cycling-related genes showed a higher occurrence in the genomes of *Alphaproteobacteria* and *Gammaproteobacteria* (Figure 2). *Bradyrhizobium* strain 78, *Mesorhizobium* strain 380, and the two *Sodalis* strains contained the nitrogenase operon *nifHDK*, which enables nitrogen fixation. Genomes of both *Sodalis* strains and *Mesorhizobium* strain 380 showed a combination of nitrogenase genes and cellulase genes. The two *Caballeronia* and the two *Sodalis* strains contained an increased number of genes for dissimilatory nitrate to nitrite reduction. Assimilatory nitrate reduction genes seem to be limited only to the presence of the *nasB* gene in a restricted number of taxa. In contrast, the assimilatory nitrite reduction genes *nirAB* were present in almost all isolated strains. Denitrification steps leading to the production of N_2_–reduction of nitric oxide and nitrous oxide–were not detected in any genome. However, several genomes (especially those in the genus *Sodalis*) contained genes for dissimilatory nitrite reduction (*nrfC* or *nrfD*), directing N flow into dissimilatory nitrate reduction to the ammonia pathway (DNRA). Similar to denitrification genes, nitrification genes, including those that allow the oxidation of ammonia, were missing in the bacterial genomes examined.

**Figure 3:**
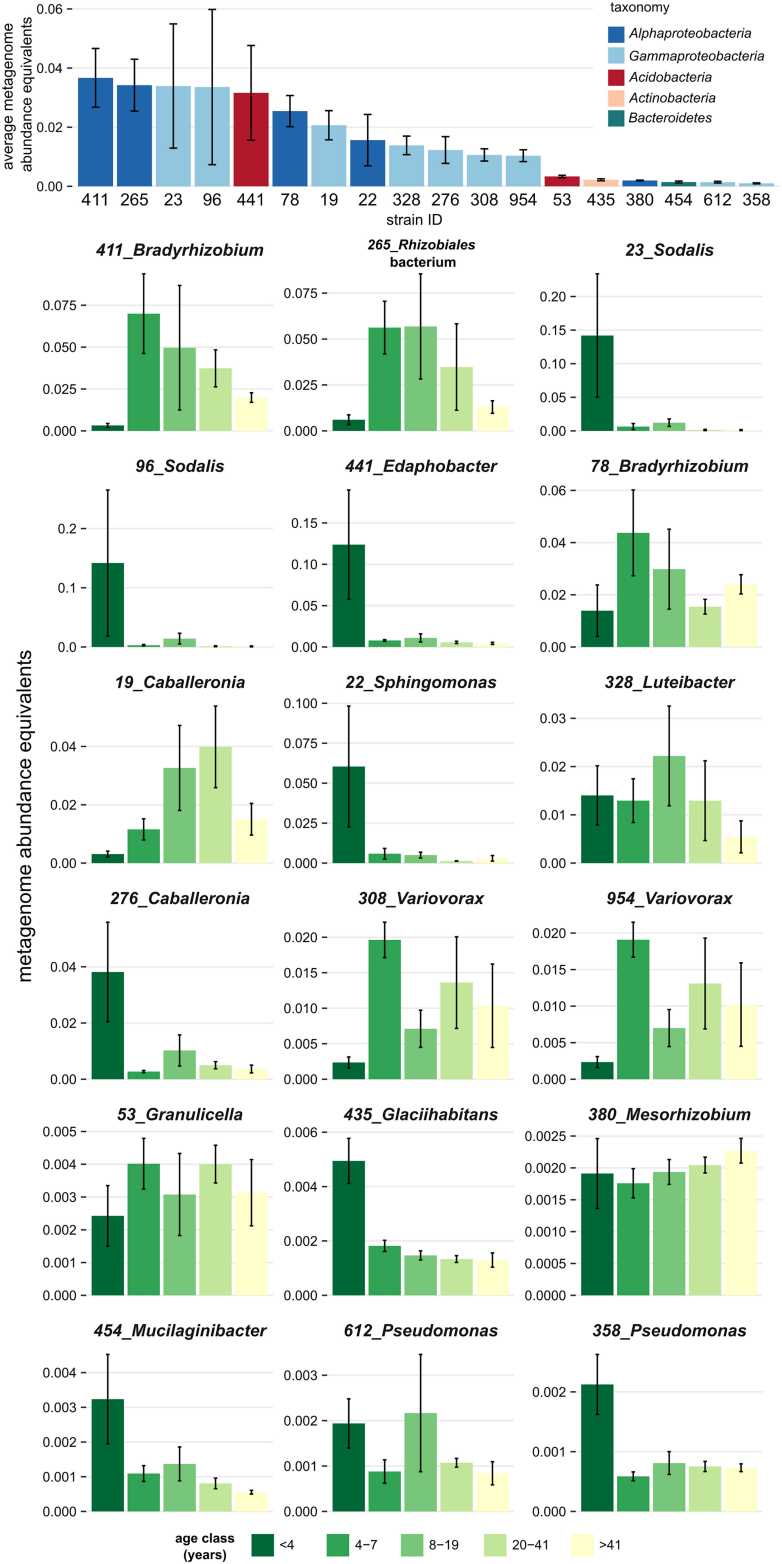
Association of dominant bacteria isolated from decomposing *Fagus sylvatica* deadwood with a certain age of deadwood. Mapping of the deadwood metagenome reads (Tláskal et al., under review) to genomes of strains shows a distinct overall abundance of these bacteria in metagenomes and distinct occurrence in individual deadwood age classes. Strains are sorted based on the total average metagenome abundance. The height of the bar plots corresponds to abundance equivalents—relative share of mapped reads corrected to the genome size of each strain. Error bars represent standard errors.

Genome-sequenced bacterial strains differed in their tendency to associate with deadwood of a certain decay length, as demonstrated by the mapping of metagenome reads from decomposing *F. sylvatica*. Mapping showed that several strains were restricted to very fresh deadwood (<4 years of decomposition), while others tended to increase in abundance at later decomposition stages (Figure 3). The isolates *Glaciihabitans* strain 435, *Pseudomonas* strain 358, *Sodalis* strains 23, and 96 and *Sphingomonas* strain 22 were significantly more abundant in the fresh CWD, while several other strains also showed high abundances in the fresh deadwood (*Edaphobacter* strain 441, *Caballeronia* strain 276 and *Mucilaginibacter* strain 454) but only inhabited certain CWD samples from the youngest age class. The abundance of other strains, including two *Bradyrhizobium* strains and *Rhizobiales* strain 265, peaked later during decomposition. Notably, the aforementioned *Alphaproteobacteria* represent bacteria with the highest abundance in the examined metagenomes (Figure 3).

## Discussion

Using prolonged cultivation, it is possible to capture a wide diversity of bacteria, including slow-growing taxa, with potentially important ecological functions (Sait et al., 2002; Lladó et al., 2016). Our isolation, which resulted in 959 deadwood bacterial isolates, was partly redundant, with many taxa repeatedly captured (Supplementary Figure 1), yet due to the presence of several members of dominant deadwood OTUs, the collection represented a relatively large portion of the bacterial community—on average, approximately one-third of the bacteria present in CWD. In contrast to culture-independent genome resolving, isolation provides complete data about the genome content of a particular strain without the threat of chimeric genomes (Shaiber and Eren, 2019) and represents a suitable approach for fully describing the role of key members of the microbiome (Valášková et al., 2009).

We characterized members of the most abundant deadwood bacterial taxa *Acidobacteria, Alphaproteobacteria, Gammaproteobacteria, Actinobacteria*, and *Bacteroidetes*. These dominant phyla appear to be generally occurring in deadwood (Johnston et al., 2016). Although our functional assignment of genes was done as accurately as possible, it should be noted that, in some cases, one CAZy family of enzymes can target different substrates (e.g., GH6; such genes found in *Glaciihabitans* displayed the highest similarity to known endo-β-1,4-glucanases). Therefore, we show the most probable functions of the CAZymes. Among cultivated bacteria, we identified strains with an increased potential for the utilization of complex carbohydrates, including cellulose, chitin, hemicelluloses, and pectin. These strains belong to the phyla *Acidobacteria, Bacteroidetes, Actinobacteria*, and the genus *Sphingomonas* from the *Alphaproteobacteria*. The strong hydrolytic capabilities of these taxa have been reported previously for some of the soil-inhabiting groups (Uroz et al., 2013; Větrovský et al., 2014; López-Mondéjar et al., 2019). Specifically, *Acidobacteria* and *Bacteroidetes* were identified as efficient decomposers of carbohydrate biopolymers (Lladó et al., 2019). This also corresponds to the identified chitinase activity (Lladó et al., 2016) and degradation of fungal mycelium by *Acidobacteria* including genus *Granulicella* (López-Mondéjar et al., 2018). This trait was supported by an increased number of chitinase genes in the cultivated *Acidobacteria* in our study.

In contrast to general degraders, deadwood *Gammaproteobacteria* and most *Alphaproteobacteria* harbored lower numbers of CAZymes, indicating a narrow spectrum of potential C sources. The least CAZy-equipped strains were those assigned to the genera *Variovorax, Pseudomonas*, and *Bradyrhizobium*. Despite the lack of CAZymes for plant-derived compound degradation, such as cellulose or hemicellulose, these low-CAZy strains have the potential to compensate for this narrow range by decomposition of fungal biomass. Adherence to fungal hyphae or enrichment in the presence of fungi were repeatedly described for *Burkholderiales*, suggesting a mycophagous lifestyle for these strains (Valášková et al., 2009; Hervé et al., 2016; Brabcová et al., 2018; Starke et al., 2020). Our *Variovorax* and *Caballeronia* strains from *Burkholderiales* indeed contained chitosanase and *N*-acetylglucosaminidase genes, respectively, which are involved in the degradation of the main fungal structural biopolymers chitin and chitosan. The genus *Pseudomonas* showed a similar chitinase gene presence to strains of the same genus involved in the decomposition of fungal biomass (Starke et al., 2020). The chitinolytic activity of the membrane-bound enzymes was measured for the *Bradyrhizobium* strains and the *Luteibacter* strain. *Luteibacter* displayed the highest chitinase activity in this study, and its mycolytic and cellulolytic activity was described previously (López-Mondéjar et al., 2016; Mieszkin et al., 2021); *Luteibacter* additionally showed a generally high activity of membrane-bound enzymes (Lasa et al., 2019).

The bacteria from the classes *Alphaproteobacteria* and *Gammaproteobacteria*, which are low in carbohydrate-active enzymes, showed the presence of genes for putative methanol oxidation. Genes for soluble diiron monooxygenases in *Caballeronia* and *Mesorhizobium* point to oxidation of other low-molecular-mass C sources rather than performing methanotrophy which was not described for these taxa (Leahy et al., 2003). Altogether, the presence of these genes suggests that *Alphaproteobacteria* and *Gammaproteobacteria* utilize simple C compounds generated during lignin decomposition by fungi (Filley et al., 2002; Lenhart et al., 2012). Methylotrophy of *Alphaproteobacteria* is sometimes accompanied by diazotrophic activity (Vorob'ev et al., 2009). Changes in bacterial deadwood community composition are tightly linked with changes in the pH, water content, and C:N ratio of the substrate (Hoppe et al., 2015; Tláskal et al., 2017). The C:N ratio of fresh deadwood is very high and decreases during decomposition, which is a result of N accumulation partially through fungal translocation and mainly through bacterial N_2_ fixation (Rayner and Boddy, 1988; Rinne et al., 2016; Tláskal et al., 2021a).

The ability to fix N_2_ might explain the association of *Alphaproteobacteria* with low-N conditions and their positive correlation with the C:N ratio (Rinta-Kanto et al., 2016; Mieszkin et al., 2021). Previous studies detected the order *Rhizobiales* (*Alphaproteobacteria*) as one of the abundant members in the deadwood community (Hervé et al., 2013) and showed the presence of nitrogenase genes assigned to this group (Hoppe et al., 2015). In line with this, we cultivated two *Rhizobiales* strains containing the nitrogenase *nifHDK* operon. The other two nitrogen fixers are enterobacteria from the genus *Sodalis*, and both are closely related to *Sodalis* insect endosymbionts that lack nitrogenase genes (Tláskal et al., 2021b). After almost 40 years, non-symbiotic N_2_-fixing *Sodalis* and *Rhizobiales* strains widen a small group of cultivated deadwood diazotrophic bacteria successfully retrieved from naturally decomposing CWD (Seidler et al., 1972; Spano et al., 1982). *Sodalis* strains were most abundant in early decomposition when the N limitation was most severe, and the abundance of *Rhizobiales* peaked later. Deadwood seems to be a hotspot of non-symbiotic N_2_ fixation, and these abundant taxa contribute to the N enrichment of the substrate (Brunner and Kimmins, 2003; Tláskal et al., 2021a). Notably, three out of four nitrogen-fixing strains (the two *Sodalis* and *Mesorhizobium* strains) also contained a single cellulase gene, which suggests that these strains might be independent in recalcitrant substrates utilization. Examination of other N cycle pathways shows that genes encoding dissimilatory steps are less common than those encoding assimilatory steps. Specifically, *Sodalis* strains (and partly *Caballeronia* strains) contain genes for DNRA, enabling the transformation of oxidized forms of N into ammonium. The balance between DNRA and denitrification is crucial for the soil N budget (Nelson et al., 2016), and a similar mechanism might exist in deadwood. The absence of complete denitrification and the preferred DNRA pathway thus prevents the loss of scarce nitrogen (Tláskal et al., 2021a).

Bacterial community composition was shown to undergo a significant change between the early and late decomposition stages at a deadwood age of ~15 years (Tláskal et al., 2017). Here, several strains showed a preference for very young and almost intact deadwood (though there was a high variation among individual fresh stems), while the same strains were less abundant in the subsequent decomposition stages. Conversely, strains that reached the highest abundance in the late decomposition phase were considerably less abundant in fresh deadwood. Bacteria involved in N_2_ fixation occur in fresh deadwood as well as in the following decomposition phases, showing the importance of this activity throughout decomposition. As the most readily available plant biopolymers are gradually depleted during decomposition, bacteria with genes for chitin degradation might switch to utilizing C from fungal biomass. Mycophagous bacteria such as *Granulicella, Luteibacter*, and *Burkholderiales* indeed tended to be more abundant in the later decomposition stages when fungal biomass in deadwood increases and starts to represent a relatively easily accessible source of both C and N.

Different C utilization strategies were described for soil habitats in which *Acidobacteria* and *Bacteroidetes* served as decomposers with a high number of expressed CAZymes, while *Proteobacteria* were recognized as opportunists relying on C uptake from other labile sources (Lladó et al., 2019). With the high taxonomic and functional overlap in these soil groups, we can identify two distinct strategies of abundant deadwood bacterial taxa: degraders with high decomposition potential and less generalist bacteria filling their C needs by alternative source utilization and mycophagy. Moreover, the latter are active in N cycling, and their presence might be beneficial for the total microbial community due to N_2_ fixation. Metabolically diverse bacteria thus show specific adaptations to challenging deadwood habitats and complement deadwood decomposition driven by fungi through N enrichment.

## Code Availability

The above methods indicate the programs used for analysis within the relevant sections. The code for reproducing the sequence processing is provided at https://github.com/TlaskalV/Deadwood-bacterial-isolates

## Data Availability Statement

Data described in this manuscript, including raw sequences from short read sequencing and genome assembly files have been deposited in NCBI under BioProject accession numbers summarized in the Table 1.

## Author Contributions

VT and PB conceived the study and wrote the manuscript. VT performed strain isolation, genome processing, and strain growth characterization. All authors contributed to the article and approved the submitted version.

## Conflict of Interest

The authors declare that the research was conducted in the absence of any commercial or financial relationships that could be construed as a potential conflict of interest.
