# Supplementary figures and images for: Deadwood-Inhabiting Bacteria Show Adaptations to Changing Carbon and Nitrogen Availability During Decomposition
Source: Front Microbiol. 2021 Jun 17;12:685303. doi: 10.3389/fmicb.2021.685303 (PMC8247643; doi:10.3389/fmicb.2021.685303)

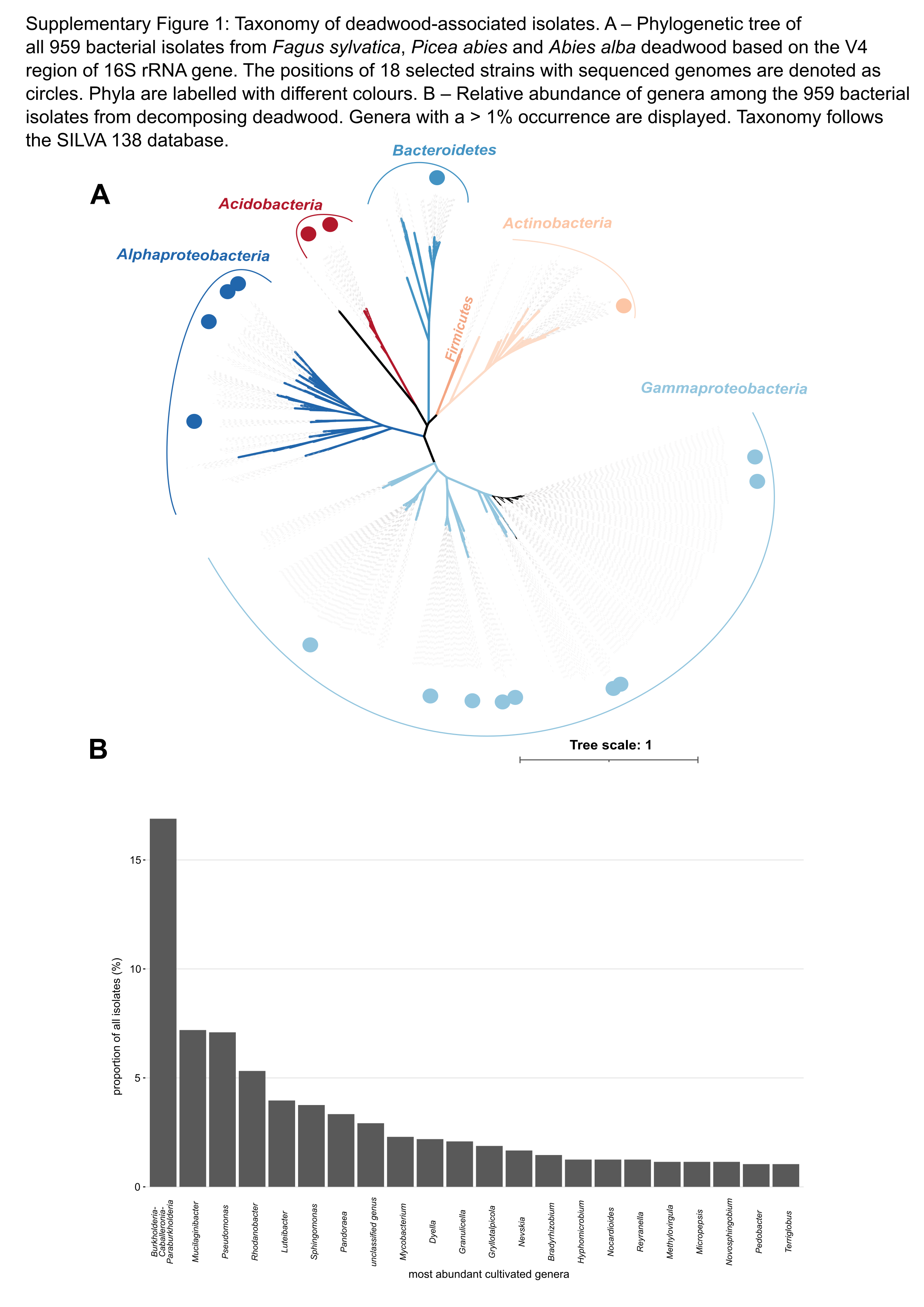

Supplement: Supplementary file 1 [file Image_1.TIFF]

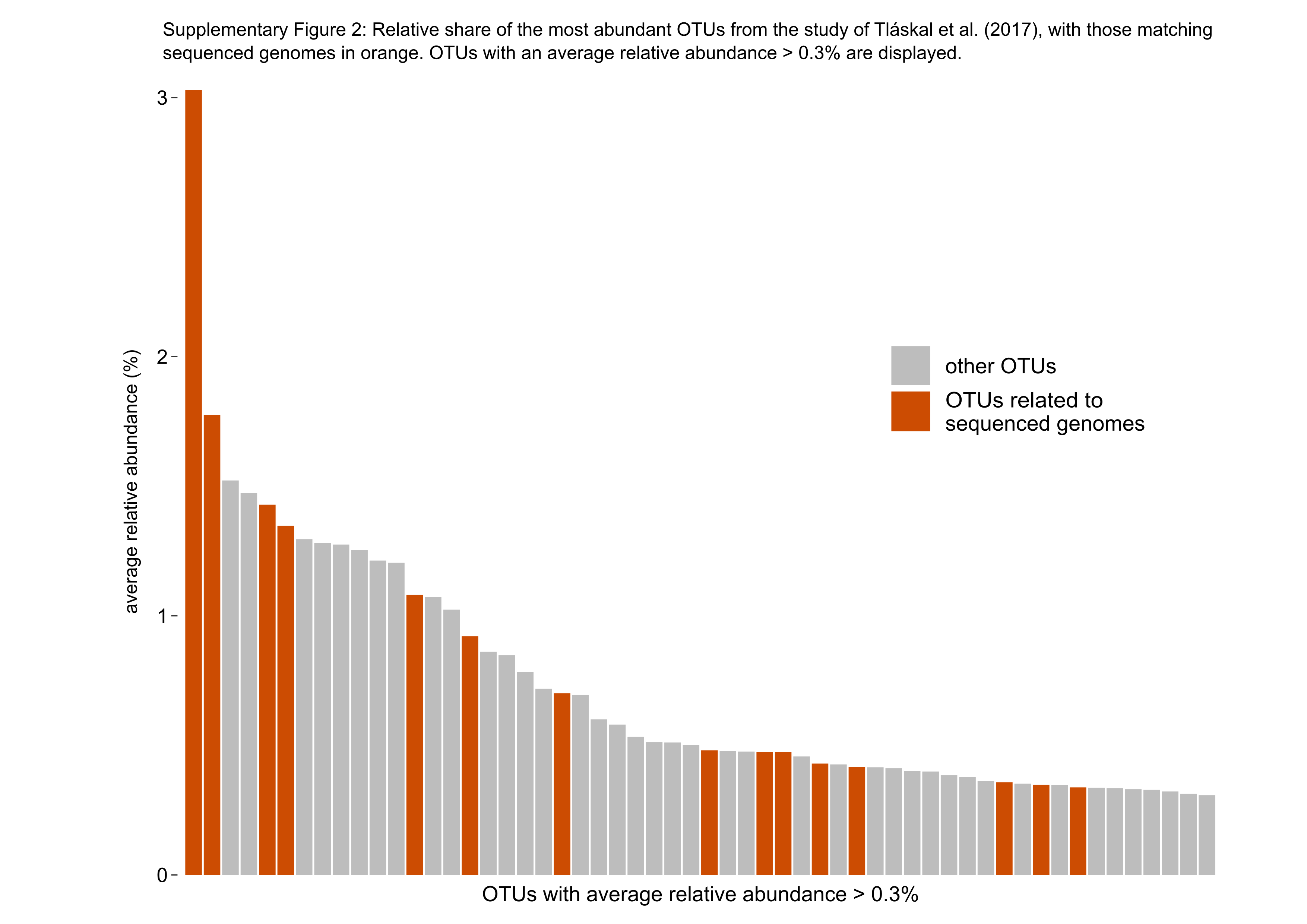

Supplement: Supplementary file 2 [file Image_2.TIFF]

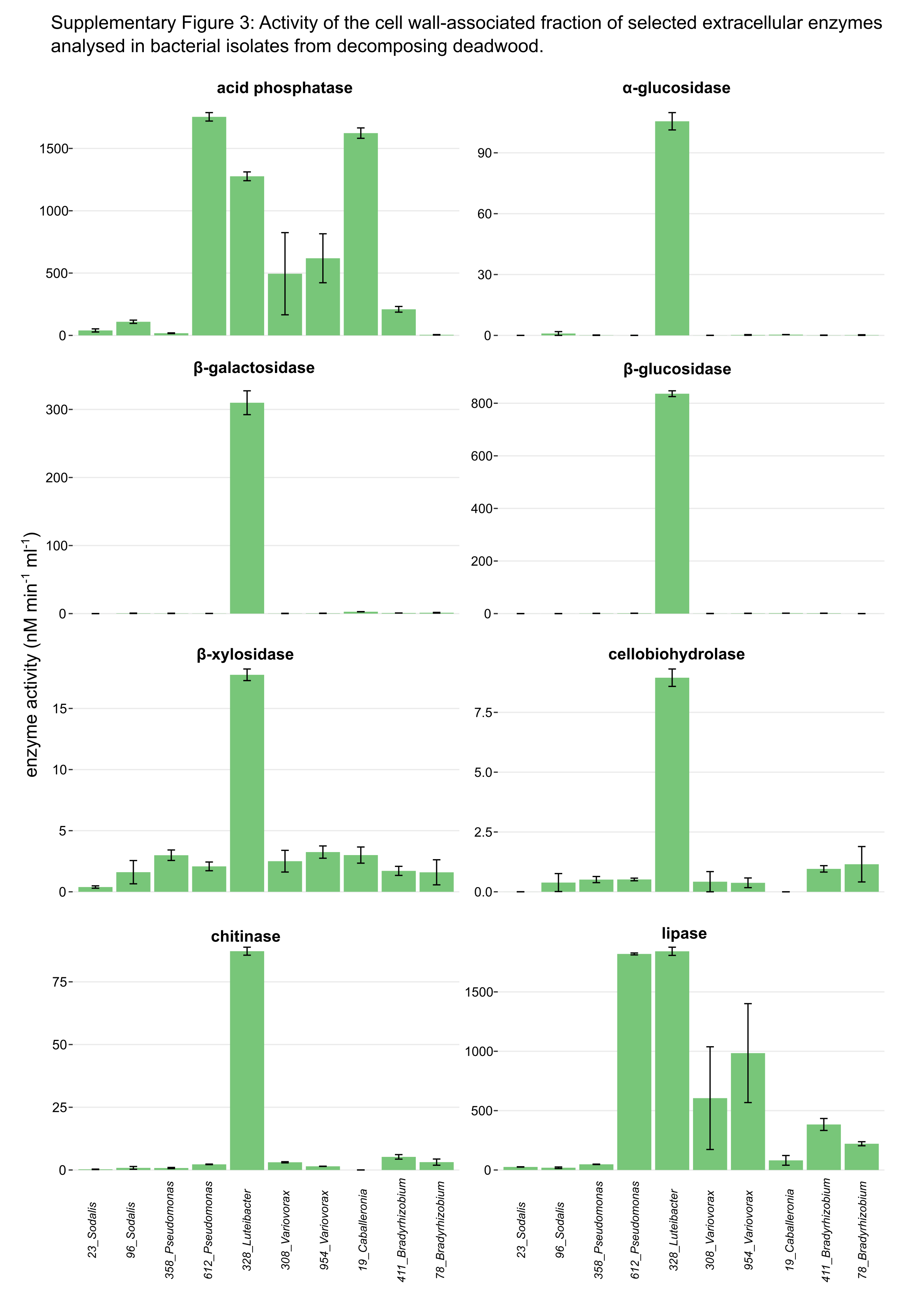

Supplement: Supplementary file 3 [file Image_3.TIFF]
